# Fatty acid binding protein 7 plays an important modulatory sex-dependent role on brain endocannabinoid levels and THC metabolism

**DOI:** 10.1371/journal.pone.0313091

**Published:** 2024-12-02

**Authors:** Samantha L. Penman, Alexandria S. Senetra, Nicole M. Roeder, Brittany J. Richardson, Ojas Pareek, Yuji Owada, Yoshiteru Kagawa, Mark S. Gold, Christopher R. McCurdy, Abhisheak Sharma, Panayotis K. Thanos

**Affiliations:** 1 Behavioral Neuropharmacology and Neuroimaging Laboratory on Addictions, Clinical Research Institute on Addictions, Department of Pharmacology and Toxicology, Jacobs School of Medicine and Biosciences, State University of New York at Buffalo, Buffalo, NY, United States of America; 2 Department of Pharmaceutics, College of Pharmacy, University of Florida, Gainesville, FL, United States of America; 3 Department of Psychology, State University of New York at Buffalo, Buffalo, NY, United States of America; 4 Department of Organ Anatomy, Graduate School of Medicine, Tohoku University, Aobaku, Sendai, Japan; 5 The Florey Institute, The University of Melbourne, Parkville, Victoria, Australia; 6 Department of Psychiatry, Washington University School of Medicine, St. Louis, MO, United States of America; 7 Translational Drug Development Core, Clinical and Translational Science Institute, University of Florida, Gainesville, FL, United States of America; 8 Department of Medicinal Chemistry, University of Florida, Gainesville, FL, United States of America; University of California Riverside, UNITED STATES OF AMERICA

## Abstract

Fatty acid binding protein 7 (FABP7) is present in the brain, but its interaction with the endocannabinoid system and phytocannabinoids is still not well understood. FABP7 has been proposed as a shuttle protein for trafficking endogenous cannabinoids, as well as an intracellular carrier of THC. In a mouse model of FABP7 global deletion, we used ultra-high performance liquid chromatography- tandem mass spectrometry (UPLC-MS/MS) to measure brain levels of Δ9 tetrahydrocannabinol (THC) as well as its primary metabolite, 11-hydroxy-THC (11-OH-THC), in male and female mice after acute inhalation of THC, compared to wild-type controls. We also measured brain levels of endogenous cannabinoids anandamide (AEA) and 2-arachidonoylglycerol (2-AG) both at baseline and after acute THC inhalation. We found that in females, brain concentrations of 11-OH-THC were significantly reduced in FABP7^-/-^ mice compared to FABP7^+/+^. Additionally, FABP7^-/-^ females had significantly reduced AEA levels and significantly increased 2-AG levels in brain tissue compared to FABP7^+/+^. Vaporized THC administration had trending, but not significant, impacts on endocannabinoid concentrations in both males and females. Our findings suggest a sex-specific role of FABP7 in the metabolism of THC as well as the regulation of endocannabinoid levels in the brain.

## 1. Introduction

The endocannabinoid (eCB) system serves as a neuromodulatory system responsible for a number of biological functions and emotional responses including cognition, appetite, sleep, and nociception [1–3]. Altered signaling of this system has been implicated in several diseases such as neurological and psychiatric disorders, substance abuse, cancer, osteoporosis, and others [2, 4–6]. The eCB system also plays a role in inflammatory pathways and immune cell development [7]. The most prominent receptors involved in this system are cannabinoid receptor 1 and cannabinoid receptor 2 (CB1 and CB2) [8].The endogenous cannabinoid 2-arachidonoylglycerol (2-AG) is a full agonist of both receptors, while the endogenous endocannabinoid anandamide (AEA) is a partial agonist of CB1 [8]. Activation of CB receptors is responsible for controlling neurotransmitter release and synaptic plasticity, largely through retrograde signaling [5, 9]. Degradation of eCBs by their catabolic enzymes (Monoacylglycerol ligase (MAGL) for 2-AG and Fatty acid amide hydrolase (FAAH) for AEA) terminates signaling [8].

Δ9-tetrahydrocannabinol (THC) is the primary pharmacological component of cannabis, responsible for a number of psychoactive and medicinal effects [10, 11]. THC is a partial agonist of both the CB1 and CB2 receptors [12] and has been shown to have a number of effects on users, ranging from hallucinations and confusion to reductions in anxiety and chronic pain [13, 14]. The primary driving force of these symptoms is CB1 receptor activation by THC [13]. THC-induced activation of the CB1 receptor is also responsible for its rewarding properties and causes reinforcing behaviors [15]. Previous studies have indicated that THC significantly reduces levels of circulating eCBs in clinical studies [15–17].

Fatty acid binding proteins (FABPs) are a group of chaperone proteins that primarily function as carriers of fatty acids throughout the cell [18]. Previous studies have indicated that FABPs may be involved in a number of addiction and drug-seeking pathways [19, 20]. There are a number of FABP subtypes, the most relevant of which are FABP3, FABP5, and FABP7, which are the primary forms of FABPs in the brain [21]. Analyses have indicated that FABPs, namely FABP5 and FABP7, are used as carrier proteins for AEA [22, 23]. The past literature has identified that the primary role of FABPs in eCB transport is their delivery to nuclear receptors or enzymes involved in eCB catabolism [23, 24]. Levels of AEA in the brain are found to be increased, and inactivation of AEA decreases upon inhibition of FABP [24, 25]. Analysis of the membrane found that an inhibitor of FABP5 and FABP7 diminished the signaling of 2-AG in the dorsal raphe nuclei neurons [24]. Additional work focused on GABA synapses has noted a reduction in tonic signaling of both AEA and 2-AG upon deletion of the FABP5 gene [26].

FABP7, also known as brain-type fatty acid binding protein, is of great interest due to its several unique characteristics and involvement in numerous physiological processes. Notably, FABP7 is not expressed in mature oligodendrocytes or neuronal cells like FABP5; instead, it is primarily found in astrocytes and neural stem cells during brain development [27]. Previous studies have also found that it is critical in maintaining neuroepithelial cells in the early stages of cortical development [28]. By adulthood, expression of FABP7 is confined to astrocytes and certain radial cells [29, 30]. A study observing the role of FABPs in the cellular transport of CBD and THC found that FABP7 has deeper binding to THC than FABPs 3 and 5 and likely serves as a carrier for both THC and CBD [31]. Additional studies indicate that FABP7 (and FABP5) bind to AEA with significantly higher affinity than FABP3 does [23].

We previously analyzed THC and metabolite serum levels in FABP5^+/+^ and FABP5^-/-^ mice and found that FABP5 is likely involved in intracellular trafficking of THC [32]. Lack of the FABP5 gene resulted in lower maximum levels of THC in serum compared to wild-type mice but resulted in higher whole-brain concentrations [32]. Deletion of FABP5 had no discernable effect on whole-brain concentrations of AEA or 2-AG, but THC did increase AEA levels regardless of genotype. In the present study, we sought to characterize the whole-brain concentrations of AEA and 2-AG both with and without THC inhalation, as well as measure THC and metabolite levels in mice lacking FABP7 proteins.

## 2. Methods

### 2.1 Animals

Male and female adult FABP7^+/+^ (on a C57BL/6J background) wild-type and FABP7^-/-^ mice were used in this study. FABP7^-/-^ breeding and genotyping were the same as previously described [33] from the original colony graciously provided by Drs. Owada and Kagawa [34]. Mice were single-housed in standard plastic cages (19 x 29 x 12 cm) in temperature-controlled conditions (22°C) on a reverse light cycle (lights off 0900–2100). Single housing was implemented to remain consistent with previous housing conditions used by our lab [32]. Food and water were provided ad libitum. All procedures conform to the National Institutes of Health Guidelines for the Care and Use of Laboratory Animals, and the protocol was approved by the Institutional Animal Care and Use Committee (IACUC) at the State University of New York at Buffalo (protocol number RIA13095Y).

### 2.2 Drugs

THC was generously provided by the NIDA Drug Supply Program. THC for inhalation was prepared in a 95% ethanol vehicle from a stock solution of 200 mg/mL as previously described, diluted to 20 mg/mL [35]. Commercially available stocks of 1 mg/mL THC, and 100 μg/mL of 11-hydroxy-THC (11-OH-THC), 11-carboxy-THC (THC-COOH), and THC-*d*3 (IS) were obtained from Cerilliant (Round Rock, TX, USA) for UPLC-MS/MS use.

### 2.3 THC treatment

Mice were placed in a sealed inhalation chamber (2.5 gallons; 16.5 x 11.3 x 5.5 cubic inch) for a 5-minute habituation period as previously described [32]. Briefly, for each exposure, four mice total were placed in the chamber simultaneously, separated by plastic dividers. THC solution was vaporized using a Volcano vaporizer (Storz and Bickel, Germany), where 0.25 mL of THC solution was dispensed onto steel pads for a final amount of 5 mg. The steel pad was placed into the vaporizer, and the drug was vaporized (226°C). Vaporized THC was collected and administered into each chamber over a 10-minute exposure period. Animals were continuously monitored throughout exposure for signs of extreme stress or labored breathing. All animals were then returned to their home cages.

### 2.4 Brain THC and eCB quantification

#### 2.4.1 Brain harvest

Mice were administered vaporized THC as previously described [32]; 15 minutes after the end of THC inhalation, mice were euthanized for brain collection. Brains were harvested from mice after euthanasia via inhaled 2% isoflurane and subsequent cervical dislocation. Brains were then flash-frozen in 2-methylbutane, weighed, and stored at −80°C prior to further work.

#### 2.4.2 Analysis of whole brains

Analysis of the whole brain was performed using a previously described UPLC-MS/MS method [32]. In short, whole mouse brains were homogenized at a 1:3 ratio with 50 mM Tris Buffer + 50 nM NF1819 (pH– 7.4 ± 0.1). Charcoal-stripped mouse plasma was utilized as a surrogate matrix and analyzed to ensure endogenous levels were < 20% of the LLOQ for analytes before preparing the calibration curve and quality controls [36]. The calibration curve consisted of THC, 11-OH-THC, THC-COOH, AEA, and 2-AG with concentrations of 1.0, 2.5, 5, 10, 50, 100, 150, and 250 ng/mL of each compound for calibration standards (CS), while quality control (QC) standards were 1.0 ng/mL (lower limit of quantification), 3.0 ng/mL (low-quality control), 125 ng/mL (median-quality control), and 225 ng/mL (high-quality control). CS, QC, and brain homogenate samples were quenched with 80 μL methanol acidified with 0.05% formic acid containing 10 ng/mL internal standard (THC-*d*3) and vortex mixed for 10 minutes. Samples were transferred to a 0.45 μm filter plate, centrifuged at 4˚C at 1500 rpm for 3 min, and filtrate was analyzed via UPLC-MS/MS.

### 2.5 Statistical analysis

All data acquired using UPLC-MS/MS were processed and quantified using the TargetLynx™ application of MassLynx™ 4.2 (Waters, Milford, MA, USA). GraphPad Prism Version 8 (GraphPad Software, San Diego, CA, USA) was used to plot each dataset and for statistical analysis (two-way ANOVA and Tukey’s multiple comparisons where applicable).

## 3. Results

**Fig 1:**
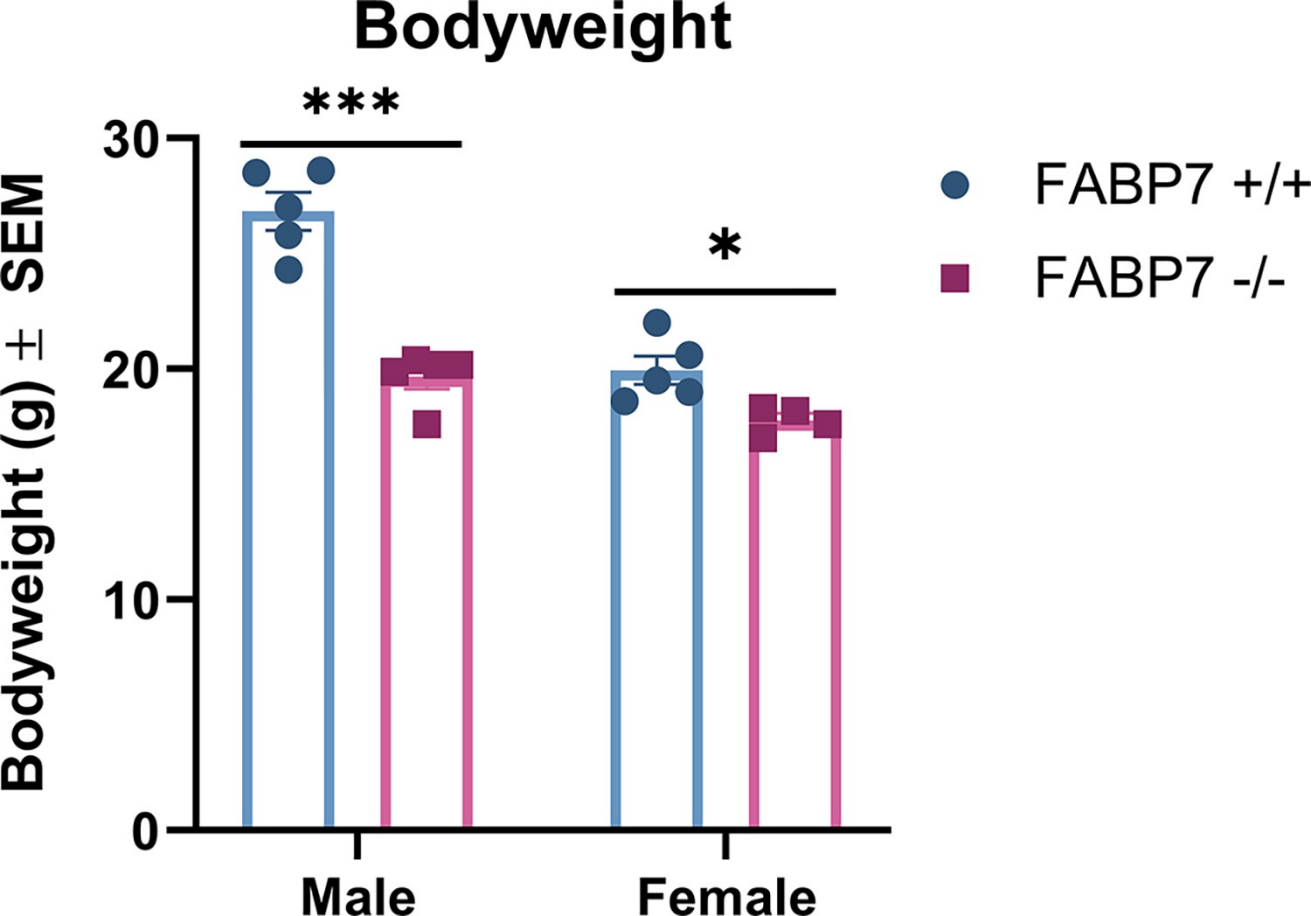
Bodyweight data for male and female FABP7^+/+^ and FABP7^-/-^ mice. Bodyweights were compared across sex and genotype. Male FABP7^+/+^ mice had significantly higher bodyweights that male FABP7^-/-^ and female FABP7^+/+^ mice. *p < .05, ***p < .001.

Bodyweights were collected immediately before THC exposure and subsequent euthanasia. A two-way ANOVA with sex and genotype as factors was run. Sex [F(1, 15) = 49.55, *p* < .001], genotype [F(1, 15) = 56.07, *p* < .001] and the interaction of sex x genotype [F(1, 15) = 16.15, *p* = .001] were all statistically significant (Fig 1). Tukey’s *post hoc* comparisons showed that male FABP7^-/-^ had significantly reduced bodyweight compared to male FABP7^+/+^ mice (*p* < .001). Female FABP7^+/+^ mice also had significantly lower bodyweight than male FABP7^+/+^ (*p* < .001).

Whole brains from FABP7^+/+^ and FABP7^-/-^ non-treated and treated (inhalation of 5 mg/pad THC) mice were collected and analyzed to determine concentrations of THC and metabolites (Table 1) along with AEA and 2-AG (Table 2). As bodyweights were statistically different, THC and metabolite levels were normalized with respect to each animal’s bodyweight to ensure any differences observed were not due to differences in weight.

**Table 1:** Brain concentrations of THC and metabolites for male and female FABP7^+/+^ and FABP7^-/-^ mice.

Brain Concentration (ng/g/kg)	Male FABP7^+/+^	Male FABP7^-/-^	Female FABP7^+/+^	Female FABP7^-/-^
THC	1102.1 ± 371.0	1591.0 ± 86.1*	2900.7 ± 1397.8	2717.9 ± 1102.2*
11-OH-THC	979.0 ± 311.2	1498.7 ± 1009.8	3158.2 ± 740.7	915.5 ± 14.9**
THC-COOH	201.5 ± 62.3**	373.1 ± 58.9**	421.0 ± 104.0	BLLOQ

Values represent the mean (N = 3–5) ± standard deviation (

*N = 4

**N = 3); values normalized to subject bodyweight.

**Table 2:** Non-treated and THC-treated brain concentrations for male and female FABP7^+/+^ and FABP7^-/-^ mice.

Brain Concentration (ng/g)	Male FABP7^+/+^	Male FABP7^-/-^	Female FABP7^+/+^	Female FABP7^-/-^
Non-treated				
AEA	47.7 ± 13.3	57.0 ± 18.8	51.4 ± 7.4	23.6 ± 4.2
2-AG	180.9 ± 36.0	209.3 ± 28.9*	180.4 ± 26.4*	287.1 ± 59.5
THC-treated				
AEA	37.2 ± 10.3	43.1 ± 13.0	39.9 ± 7.5	31.9 ± 7.1*
2-AG	173.1 ± 42.3	189.0 ± 39.1	174.1 ± 20.0	242.5 ± 33.6*

Values represent the mean (N = 4–5) ± standard deviation (

*N = 4).

**Fig 2:**
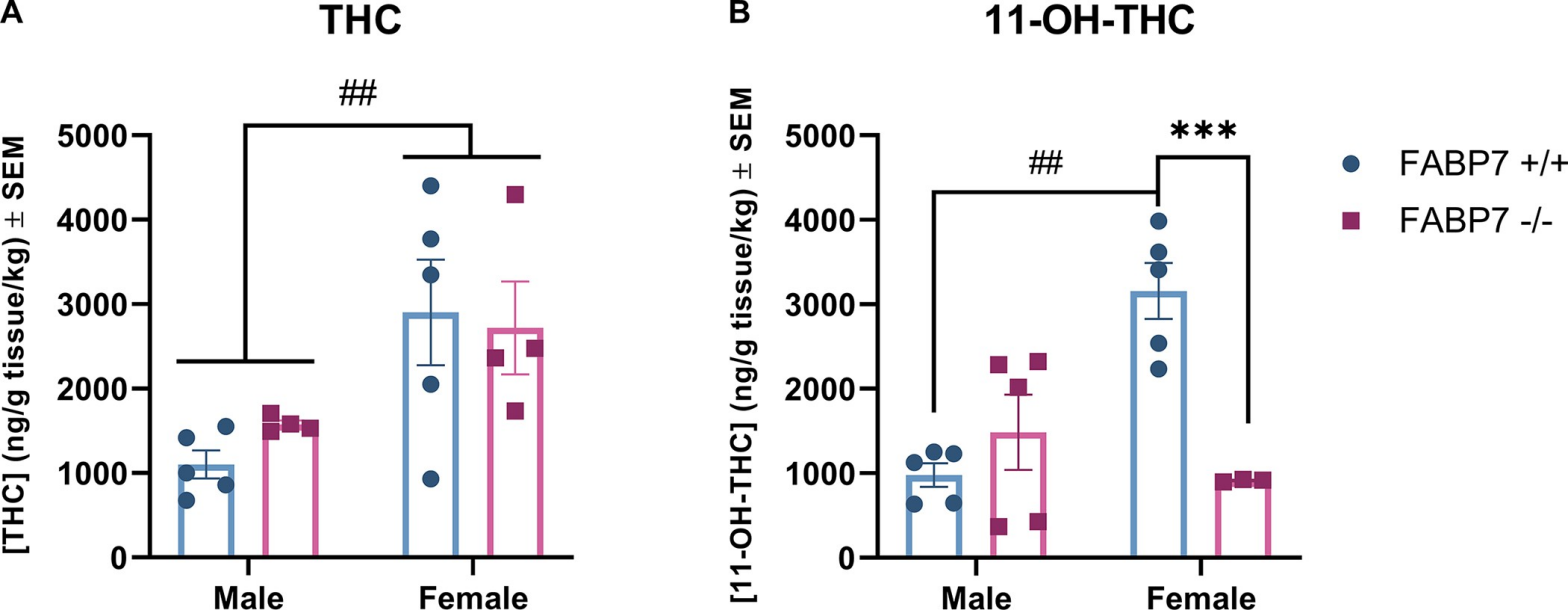
Analysis of exogenous cannabinoids in the brain. Concentration of exogenous cannabinoids in whole brain samples following a 10 min inhalation dose of 5 mg/pad THC in FABP7^+/+^ and FABP7^-/-^ mice, normalized to subject bodyweight. (A) Females had greater levels of THC in the brain compared to males, regardless of genotype. (B) FABP7^+/+^ females had increased 11-OH-THC levels compared to FABP7^+/+^ males. FABP7^-/-^ females had reduced 11-OH-THC levels compared to FABP7^+/+^ females. #*p*<0.05 main sex effect, ##*p*<0.01 sex effect, **p<0.01 genotype effect.

For analysis of THC and 11-OH-THC, two-way ANOVAs with sex and genotype were used as factors. THC-COOH could not be analyzed as all FABP7^-/-^ females had levels BLLOQ (Below Lower Limit of Quantification). Analysis of THC showed that there was a significant main effect of sex on THC levels in the brain following acute inhalation [F (1, 14) = 11.06, *p* = 0.0050], but there was no effect of genotype (Fig 2A). Analysis of primary metabolite 11-OH-THC showed a significant main effect of genotype [F (1, 14) = 6.686, *p* = 0.0216] and sex [F (1, 14) = 5.736, *p* = 0.0312] as well as a significant interaction of sex and genotype [F(1, 14) = 17.18, *p* = 0.001]. Post hoc analysis with Tukey’s multiple comparisons showed that female FABP7^+/+^ mice had significantly greater levels of 11-OH-THC in the brain compared to male FABP7^+/+^ (*p* = 0.0012); additionally, female FABP7^-/-^ mice had significantly lower levels of 11-OH-THC compared to FABP7^+/+^ females (*p* = 0.0033) (Fig 2B).

**Fig 3:**
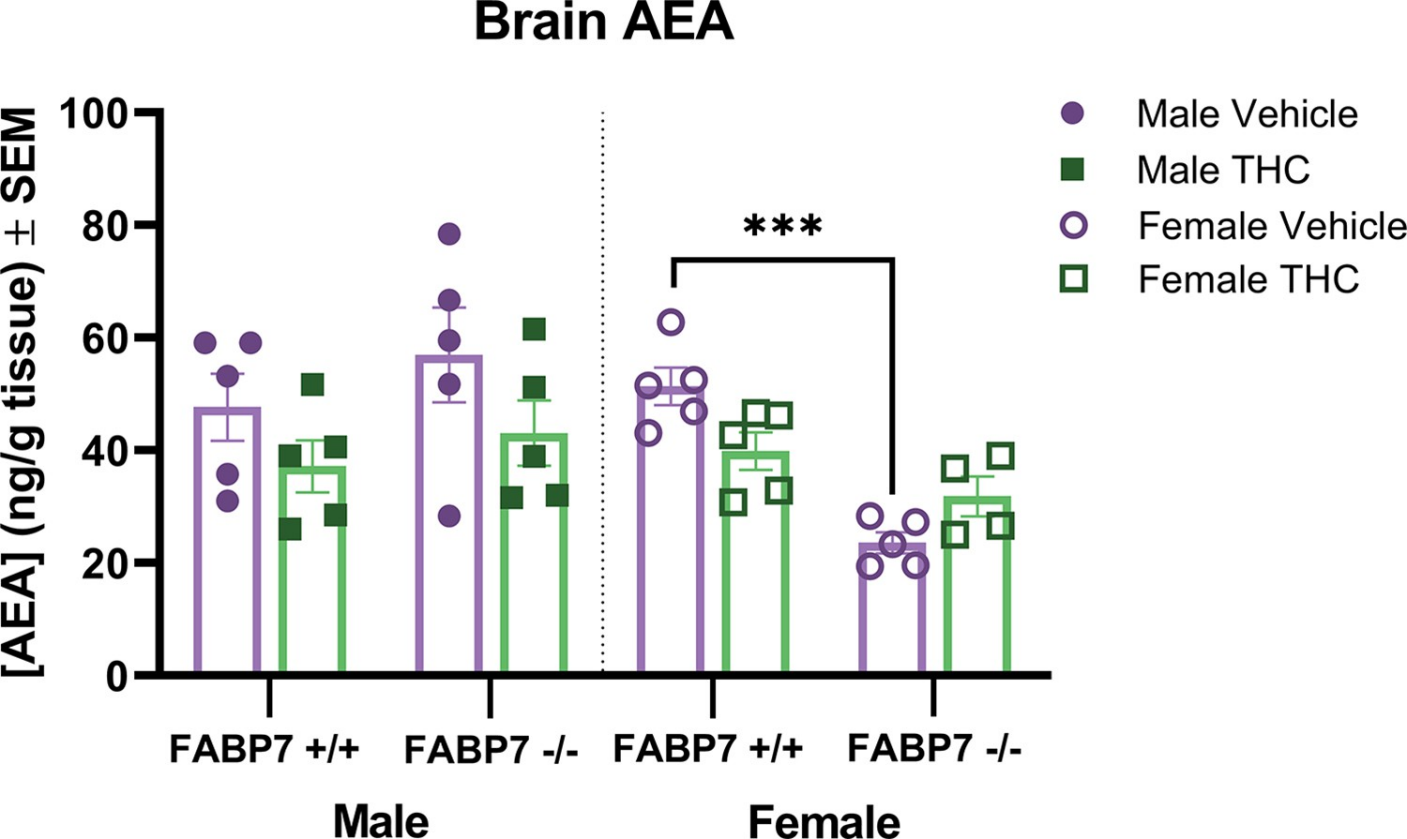
Analysis of anandamide in the whole brain. Concentrations of endogenous cannabinoid AEA in whole brains following vehicle or a 10 min inhalation dose of 5 mg/pad THC in FABP7^+/+^ and FABP7^-/-^ mice. Males showed a trending effect of treatment where THC treatment reduced AEA levels regardless of genotype. Female FABP7^+/+^ mice treated with THC showed trending reduction in brain AEA compared to vehicle-treated. Vehicle FABP7^-/-^ female mice had significantly lower brain AEA levels compared to FABP7^+/+^ females. ***p<0.001 genotype effect.

For analysis of AEA and 2-AG levels, two-way ANOVAs with genotype and treatment were run for each sex. For AEA levels, males showed no significant effect of genotype or THC treatment. In females, there was a main effect of genotype [F (1, 15) = 33.97, *p* < .001] as well as an interaction of genotype and drug treatment. Post hoc analysis with Tukey’s multiple comparison test showed a significant reduction in AEA for FABP7^-/-^ compared to FABP7^+/+^ after vehicle treatment (*p*<0.001) (Fig 2). Analyzing 2-AG levels found that males showed no significant effect of genotype or treatment, while females showed a significant main effect of genotype [F (1, 14) = 22.49, *p*<0.001] (Fig 3).

## 4. Discussion

**Fig 4:**
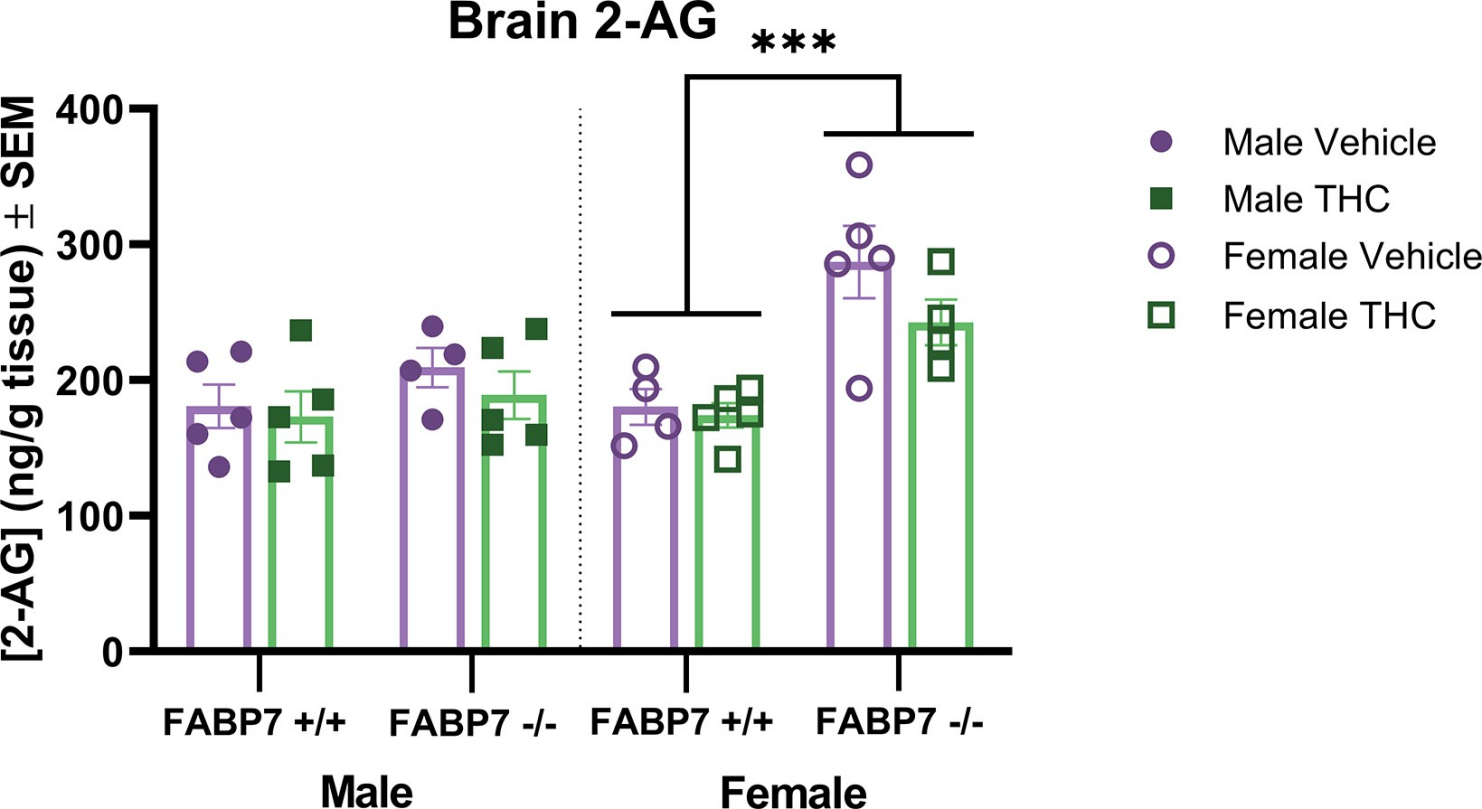
Analysis of 2-arachidonylglycerol in whole brain. Concentrations of endogenous and exogenous cannabinoids in whole brains following vehicle or a 10 min inhalation dose of 5 mg/pad THC in FABP7^+/+^ and FABP7^-/-^ mice. FABP7^-/-^ females had increased 2-AG levels in the brain compared to FABP7^+/+^ females regardless of treatment. ***p<0.001 main genotype effect.

Our results in the present study indicate a complex relationship between FABP7, exogenous cannabinoid administration, and sex. Females showed increased levels of THC in the brain regardless of genotype (Fig 2). When looking at the primary metabolite (11-OH-THC), this sex difference persisted in wild-type subjects but was not present with FABP7 deletion (Fig 2B). Males overall exhibited a trending reduction in AEA brain levels following acute THC administration, regardless of genotype. Females with ablated FABP7 had significantly reduced levels of AEA compared to FABP7^+/+^ subjects under vehicle conditions; acute THC administration resulted in a trending reduction of AEA levels in FABP7^+/+^ females as males exhibited but had no discernable effect on FABP7^-/-^ females (Fig 3). 2-AG levels were largely unaffected by acute THC administration, but FABP7^-/-^ females overall had increased brain levels compared to FABP7^+/+^ counterparts (Fig 4).

Sex differences in response to THC administration have been extensively described in the literature in both human [37–41] and rodent studies [42–48]. Females in preclinical studies have been shown to metabolize THC differently, wherein females have higher levels of primary metabolite 11-OH-THC than males [45, 49, 50], likely due to differences in metabolizing enzyme CYP450 levels [51, 52]. Interestingly, this can coincide with similar levels of THC between sexes in serum and brain tissue [45] or even lower levels of THC in serum and brain tissue [53]. Our results showed higher levels of THC in the brain for females compared to males, regardless of genotype (Fig 2). A recent study in B6J mice that examined acute cannabis smoke exposure found that while male mice exhibited greater plasma concentrations of CBD 10-minutes post-exposure, when measuring brain levels of THC there was no difference across sexes [54]. This difference may be due to the differences in the inhalation paradigms used- the study from Gazarov and colleagues used cannabis cigarettes as opposed to THC stock, and inhalation occurred over 1 hour rather than 10 minutes. A previous inhalation study in rats found that in Wistar rats, there was a sex difference where females had higher THC serum levels than males, but this was not present in Sprague Dawley rats [55]. This may indicate an important interaction between sex and species/strain on THC pharmacokinetics. As THC has been shown to rapidly absorb into fat and remain stored for long periods [56–58], it is possible the males may have reduced THC measured in the brain due to fat sequestration, especially as the FBAP7^+/+^ males had significantly increased bodyweight (Fig 1). Clinically, sex differences have been observed in the development and pathology of substance use disorders including cannabis, alcohol, opiates, and cocaine [40, 41, 59–61], but mechanisms behind these sex differences are still not fully understood. While we saw no effect of genotype in the THC brain concentrations, we found that deletion of FABP7 in females significantly reduced 11-OH-THC brain levels compared to wild-type (Fig 2). Elmes and colleagues previously identified FABP1 as an important mediator of THC metabolism in the liver [62], but there is little work investigating the interaction of FABP7 and THC in the liver. FABP7 expression in the liver is thought to be limited to Kupffer cells, permanent macrophages involved in various processes depending on normal or pathological conditions [63, 64]. In states of liver injury, FABP7 is implicated in the regulation of phagocytosis and cytokine production [64]. Our results indicate that there may be a role of FABP7 in the liver in THC metabolism in a sex-dependent manner, but further work is needed to fully elucidate this. Interestingly, we previously found no indication of FABP5 deletion impairing the liver metabolism of THC [32].When examining endogenous cannabinoid levels, we found that males no change in AEA following THC administration, regardless of genotype (Fig 3). FABP7^+/+^ females also exhibited no change in AEA levels across treatment. Previous work has shown that THC administration may result in increased AEA levels. A 2015 study suggests that this may be due to competition between exogenous cannabinoid THC and endogenous AEA for FABP binding [31]. Interestingly, we also recently found increased AEA levels following the same THC inhalation paradigm in B6N mice, both wild-type and FABP5^-/-^ [32]. As all conditions between our previous work and the present study are identical other than mouse strain (B6N vs. B6J), this could be due to a metabolic difference between the substrains [65, 66]. The FABP7^-/-^ females had significantly reduced AEA compared to FABP7^+/+^ under baseline conditions, and there was no discernable effect of THC administration (Fig 3). Previous studies investigating the interaction of estradiol with the endocannabinoid system have found that estradiol mediates FAAH expression, the enzyme responsible for AEA degradation [67–69]. Estradiol has been shown to increase AEA release in endothelial cells and in the uterus [68, 69]. Estradiol may also mediate AEA synthesis in the brain through an ERα-mGLuR1 pathway in females [70]. Additionally, females have been shown to exhibit tonic AEA signaling, unlike males [70–72]. It is possible there is a relationship between FABP7 deletion and estrogen levels mediating the reduced AEA levels in the FABP7^-/-^ females at baseline, but more investigation is needed to parse this possible relationship out.

Acute THC administration had no effect on 2-AG levels in the brain, and there were no differences in males across genotypes. Females, regardless of THC treatment, had increased 2-AG levels with FABP7 deletion (Fig 4). There are not many studies examining sex differences in 2-AG content in the brain. One study in Wistar rats found that compared to males, females had decreased expression of MAGL, the enzyme responsible for 2-AG catabolism, in select brain regions [73]. This, in theory, would result in increased levels of 2-AG in females compared to males. Another study in Sprague Dawley rats found that while females showed reduced 2-AG in the periaqueductal grey, there was increased 2-AG measured in the trigeminal nucleus caudalis and occipital V1M cortex compared to males [74]. A study from 2019 investigating the influence of sex on eCB tone at birth found that injections of testosterone, but not estradiol, critically regulated 2-AG levels, increasing 2-AG after administration in female Sprague Dawley pups on postnatal day 0 and 1 [75]. Additionally, females at birth have shown lower brain levels of both 2-AG and AEA [76]. This may indicate that the increased levels of 2-AG we have observed in FABP7^-/-^ females may be due to a critical role of FABP7 during female development in the endocannabinoid system.

Our study is not without limitation. We recognize the confound of single housing used in this study, which can alter baseline endocannabinoid tone [77, 78]. An additional limitation inherent to the design is the inability to dose for bodyweight in an inhalation paradigm. This is somewhat accounted for by normalizing the data of exogenous cannabinoid levels to bodyweight. Differences in the respiratory rates could be measured in future work through use of metabolic cages, and is worthwhile to investigate.

The present results indicate a complex relationship between FABP7, endocannabinoid signaling, and THC metabolism in female mice. The effects of FABP7 deletion observed here may have roots in gestational development, as there is evidence of eCB system development being regulated by sex hormones during gestation [75, 76]. We previously did not observe sex differences in eCB levels or THC in the brain following FABP5 deletion [32]. Estradiol influence in gestation differs in males and females, defeminizing genetic males during gestation and feminizing genetic females postnatally [79]. Future work examining THC metabolism and eCB levels in the brain at different stages of estrous is of interest, as eCBs fluctuate during human estrous cycling as well [80]. There may be a FABP7–estradiol relationship in development that influences eCB tone in adulthood, but further work will need to be done to fully understand our results. Future work should also look to analyze AEA and 2-AG levels in an FABP7 global deletion model across different brain regions, as past studies have indicated these levels can be region dependent [81]. The eCB system has attracted increasing interest in recent years as a possible therapeutic target for many diseases and ailments ranging from nausea to epilepsy to nicotine addiction, with very few successes so far [2, 82]. Chronic stimulation of the eCB system through repeated use of cannabis, particularly in development, has shown increased risk of cognitive deficits and comorbid psychiatric disorders [83, 84]. Our results may indicate FABP7 as a potential target of interest for future research, particularly in females.
